# On the Estimation of Intron Evolution

**DOI:** 10.1371/journal.pcbi.0020084

**Published:** 2006-07-28

**Authors:** Miklós Csűrös

*PLoS Computational Biology* recently published an article about spliceosomal intron evolution by Nguyen, Yoshihama, and Kenmochi [1]. The authors were unaware of some earlier independent results. Foremostly, the main point of the article—that of estimating the density of potential intron sites—is not novel. It was described more than three months earlier [2]. The numerical results are virtually identical in the two publications, which is not surprising, since they apply the same model to the same data [3]. A recent article points to the model's validity. Raible and coauthors [4] report that introns in the protostome Platynereis dumerilii are almost as abundant as in humans, and many introns are in homologous positions between the two species. The shared positions indicate that at most one-third of human introns were gained in the vertebrate lineage, in agreement with the estimates of [2] and [1]. In contrast, parsimony estimates [3] should change significantly when including P. dumerilii.

To estimate ancestral intron losses and gains, Nguyen and coauthors use an exponential-time procedure, which is practical only for a few species. In reality, the estimation can be done in linear time [2], as described briefly below. We are modeling intron presence and absence in homologous sites across organisms related by a known phylogeny. Presence and absence are encoded by 1 and 0, respectively. Introns evolve independently, by a Markov model for a binary character. On branch *e,* an intron is lost with probability *p_e_*(1 → 0) and an intron is gained with probability *p_e_*(0 → 1) at every site. Assuming a continuous-time Markov process,

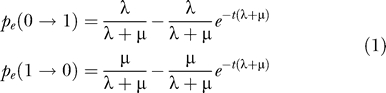
where *λ, μ* > 0 are branch-specific gain and loss rates, and *t* > 0 is branch length. Introns are observed at the terminal taxa. An all-absent intron site is never observed, and, thus, the number of potential intron sites must be estimated for correct likelihood optimization. The likelihood can be computed by a dynamic programming algorithm [5]. The algorithm calculates the *conditional likelihood L_u_*(*x*) for every node *u* and state *x* ∈ {0,1}: *L_u_*(*x*) is the probability of the observed states in descendants of *u,* conditioned on the state *x* at *u*. One can further define the *upper conditional likelihood U_u_*(*x*) for the observed states *outside* the subtree of *u,* which can be computed efficiently by dynamic programming even if the underlying process is irreversible [2]. Feslenstein [6] reviews relevant techniques for the reconstruction of ancestral molecular sequences, which are generally assumed to evolve by a reversible process. Now, the posterior probability of the intron state *x* at every node *u* can be computed as


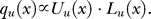


The posterior probability for state change *x* → *y* on an edge *uv* is computed as


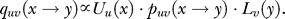


The expected numbers of gains or losses are obtained by summing the probabilities *q_uv_*(0 → 1) and *q_uv_*(1 → 0) over all intron sites, respectively. Nguyen and coauthors consider instead all 2*^N^* state labeling of *N* internal nodes to compute the expected numbers of gains and losses. A Java package implements the more efficient procedure, and is publicly available at http://www.iro.umontreal.ca/~csuros/introns/.

Nguyen et al. [1] reiterate well-known concerns of identifiability. Their Proposition 1 echoes the Pulley Principle for ambiguous root placement [5]. Proposition 2 asserts that there are two possible parameter sets *p_e_*(*x* → *y*) for every branch, which can be combined to get exponentially many choices that give the same likelihood function. The continuous-time process of Equation 1 implies *p_e_*(0 → 1) + *p_e_*(1 → 0) < 1. Such constraint leads to unique parametrization (except for the root position), and is more natural than the one proposed by Nguyen and coauthors, which is based on the variance of intron gains and losses.

Nguyen and coauthors discuss an important study by Qiu, Schisler, and Stoltzfus [7]. Qiu and coauthors constructed multiple alignments of ten gene families. The families had 68 sequences and 49 intron sites on average. Using a Bayesian framework, Qiu and coauthors estimated two intron evolution parameters per family, assuming constant rates across sites and branches. The model's adequacy and some of the conclusions can certainly be debated, especially in view of the assumption of constant rates. Nguyen and coauthors, however, speculate that the data were insufficient for valid inference, since there are 2^68^ possible intron presence–absence patterns for the average gene family, but only 49 intron sites. The argument is not sound: the number of patterns has little to do with inference (consider the case of a protein alignment with 20*^k^* possible patterns for *k* sequences). It is the number of parameters that matters. 
